# The Absence of NOD1 Enhances Killing of *Aspergillus fumigatus* Through Modulation of Dectin-1 Expression

**DOI:** 10.3389/fimmu.2017.01777

**Published:** 2017-12-13

**Authors:** Mark S. Gresnigt, Martin Jaeger, R. K. Subbarao Malireddi, Orhan Rasid, Grégory Jouvion, Catherine Fitting, Willem J. G. Melchers, Thirumala-Devi Kanneganti, Agostinho Carvalho, Oumaima Ibrahim-Granet, Frank L. van de Veerdonk

**Affiliations:** ^1^Unité de recherche Cytokines and Inflammation, Institut Pasteur, Paris, France; ^2^Laboratory for Experimental Internal Medicine, Department of Internal Medicine, Radboud University Medical Center, Nijmegen, Netherlands; ^3^Department of Immunology, St. Jude Children’s Research Hospital, Memphis, TN, United States; ^4^Unité Histopathologie Humaine et Modèles Animaux, Département Infection et Epidémiologie, Institut Pasteur, Paris, France; ^5^Department of Medical Microbiology, Radboud University Medical Centre, Nijmegen, Netherlands; ^6^Life and Health Sciences Research Institute (ICVS), School of Medicine, University of Minho, Braga, Portugal; ^7^ICVS/3B’s – PT Government Associate Laboratory, Braga/Guimarães, Portugal

**Keywords:** NOD1, *Aspergillus fumigatus*, nucleotide-binding oligomerization domain, dectin-1, fungal killing

## Abstract

One of the major life-threatening infections for which severely immunocompromised patients are at risk is invasive aspergillosis (IA). Despite the current treatment options, the increasing antifungal resistance and poor outcome highlight the need for novel therapeutic strategies to improve outcome of patients with IA. In the current study, we investigated whether and how the intracellular pattern recognition receptor NOD1 is involved in host defense against *Aspergillus fumigatus*. When exploring the role of NOD1 in an experimental mouse model, we found that *Nod1^−/−^* mice were protected against IA and demonstrated reduced fungal outgrowth in the lungs. We found that macrophages derived from bone marrow of *Nod1^−/−^* mice were more efficiently inducing reactive oxygen species and cytokines in response to *Aspergillus*. Most strikingly, these cells were highly potent in killing *A. fumigatus* compared with wild-type cells. In line, human macrophages in which NOD1 was silenced demonstrated augmented *Aspergillus* killing and NOD1 stimulation decreased fungal killing. The differentially altered killing capacity of NOD1 silencing versus NOD1 activation was associated with alterations in dectin-1 expression, with activation of NOD1 reducing dectin-1 expression. Furthermore, we were able to demonstrate that *Nod1^−/−^* mice have elevated dectin-1 expression in the lung and bone marrow, and silencing of *NOD1* gene expression in human macrophages increases dectin-1 expression. The enhanced dectin-1 expression may be the mechanism of enhanced fungal killing of *Nod1^−/−^* cells and human cells in which NOD1 was silenced, since blockade of dectin-1 reversed the augmented killing in these cells. Collectively, our data demonstrate that NOD1 receptor plays an inhibitory role in the host defense against *Aspergillus*. This provides a rationale to develop novel immunotherapeutic strategies for treatment of aspergillosis that target the NOD1 receptor, to enhance the efficiency of host immune cells to clear the infection by increasing fungal killing and cytokine responses.

## Introduction

Invasive aspergillosis (IA) is an opportunistic fungal infection that globally affects hundreds of thousands severely immunocompromised patients on an annual basis (1). IA is associated with an unacceptable high mortality (2), yet modern antifungal drugs, patient isolation care, and prophylactic treatment strategies have not been able to reduce mortality over the past years. An increased knowledge of the antifungal host response is crucial for understanding the pathogenesis of the disease on one hand and on the other hand in the development of new immunomodulatory therapies, which are considered to be one of the few possibilities to decrease mortality associated with IA (3).

A fine-tuned interplay between recognition and signaling leads to the clearance of the fungus by the immune system, while defects in parts of these components or their absence have been associated with severe infections with the fungus. Although most types of PRRs, including toll-like receptors and C-type lectin receptors, have well-characterized roles in antifungal host defense (4, 5). Some PRRs have, however, not yet been evaluated for their role in antifungal host defense. Insights in these not yet explored PRRs might yield new insights in the pathogenesis of IA and provide potential candidate targets for novel treatment strategies.

The nucleotide-oligomerization domain (NOD) receptors play a crucial role in host defense against bacteria; however, only limited evidence is available regarding the role of these receptors in host defense against aspergillosis. One of the NOD receptors, NOD1, has been described to be able to activate NFκB in corneal epithelial cells in response to *Aspergillus fumigatus* (6). However, it is not yet investigated whether NOD1 plays a role in host defense against pulmonary aspergillosis. Overall, it is evident that NOD1 plays an important role in pulmonary host defense. NOD1 is highly expressed in the lung (7) and in lung epithelial cells (8). Human alveolar macrophages utilize NOD1 to induce proinflammatory cytokine responses and induce autophagy for an efficient host defense against *Mycobacterium tuberculosis* (9). Moreover, in host defense against *Legionella pneumophila*, NOD1 regulates neutrophil recruitment to the alveoli (10, 11). These studies of pulmonary host defense against bacteria reveal various mechanisms, induced by NOD1, that are known to play significant roles in host defense against *A. fumigatus*; e.g., autophagy machinery (12–15), neutrophil recruitment (16–18), and proinflammatory cytokines (19–21).

Therefore, the current study investigates the role of NOD1 in host defense against aspergillosis. Specifically, in a murine model representing immunocompromised hosts, we assess how NOD1 deficiency influences the host defense during aspergillosis. Using murine *Nod1*-deficient cells as well as silencing of *NOD1* gene expression in primary human cells, we systematically evaluated the importance of this receptor in the antifungal response. Novel insight into the exact biology of this receptor during aspergillosis can increase our understanding of the infection, which subsequently may lead to the development of immunotherapeutic strategies.

## Materials and Methods

### *Aspergillus* *fumigatus*

A clinical isolate of *A. fumigatus* V05-27, which has been characterized previously (22), was used for all *ex vivo* and *in vitro* stimulations. Conidia and hyphae were prepared and heat-inactivated (HI) as previously described (23). A concentration of 1 × 10^7^/mL was used in the experiments unless otherwise indicated. For *in vivo* experiments, the luciferase-expressing *A. fumigatus* 2/7/1 strain was used, which has been described previously (24); this strain has been reported to have a similar antifungal susceptibility and demonstrates no growth defects under various *in vitro* cultivation conditions such as different temperatures and carbon sources (24). In corticosteroid immunosuppressed mouse models of aspergillosis (25), the 2/7/1 strain demonstrated a similar virulence as observed for its parental strain CBS144.85 (26, 27).

### *In Vivo* Experiments

Mice for *in vivo* experiments were supplied by the breeding center R. Janvier (Le Genest Saint-Isle, France). For the survival experiment in an immunosuppressed background C57/BL6 wild type (WT), and *Nod1^−/−^* mice (28 to 31 g, 10 weeks old) were used. Mice were immunosuppressed at day 4 and day 1 before infection by intraperitoneal injection of 200-µL cyclophosphamide (Sigma Aldrich) at 4 mg/mL. At the day of infection, mice were anesthetized by intramuscular injection (150 µL) of ketamine (10 mg/mL) and xylasine (10 mg/mL) hair was shaved from the ventral lung area and subsequently mice were inoculated intranasally with 5 × 10^4^ luciferase-expressing *A. fumigatus* 2/7/1 conidia (24) in 25-µL PBS.

In all experiments, survival and weight was monitored during the course of infection. Bioluminescence imaging was acquired at day 1 post-infection (pi) and was continued on days 2, 3, 6, and 8 pi. Images were acquired using an IVIS 100 system (PerkinElmer, Waltham, MA, USA) as previously described (25).

For immunological and histological assessment female C57/BL6 and *Nod1^−/−^* mice (19–22 g, 8 weeks old) were used. They received similar immunosuppression regimen and were similarly infected as the mice for survival. Weight and bioluminescence were monitored daily during the course of infection. At day 3, the mice were euthanized. Serum and BAL were collected and lung homogenates were obtained following disruption in saline using the Retsch Mixer Mill 301 homogenizer. Cytokine concentrations in BAL and plasma were determined by ELISA as specified by the manufacturer (DuoSet; R&D Systems).

The fungal burden was determined by amplification of *Aspergillus* ITS2 regions. Briefly, homogenized tissue samples were used for DNA isolation by using the automated MagNA Pure system and the MagNA Pure LC Total Nucleic Acid Isolation Kit according to manufacturer’s protocol (Roche Applied Science). PhHV was added to all samples as an internal isolation control C.

The concentration of total isolated DNA was measured by using the Quantus Fluorometer (Promega). *Aspergillus* loads were determined by real-time PCR using the LC480 instrument and the probes master kit (Roche applied Science). Thermocycling conditions were as follows: 37°C for 10 min, 95°C for 10 min, and 50 cycles: 95°C for 15 s, and 60°C for 45 s. The rDNA ITS2 region of *A. fumigatus* was detected by using primers 5′-GCGTCATTGCTGCCCTCAAGC-3′, 5′-ATATGCTTAAGTTCAGCGGGT-3′ and probe Cy5-TCCTCGAGCGTATGGGGCTT-BBQ. The PhHV isolation control was detected by using primers 5′-GGGCGAATCACAGATTGAATC-3′, 5′-GCGGTTCCAAACGTACCAA-3′ and probe LC610-TTTTTATGTGTCCGCCACCATCTGGATC- BBQ. For the ITS2 detection, a twofold dilution series of the cloned PCR product was included to calculate the number of copies per reaction.

### PBMC Isolation and Stimulation

Venous blood samples from healthy controls and patients were obtained after written informed consent. PBMCs were isolated as previously described (23). Briefly, blood was diluted in PBS (1:1) and fractions were separated by Ficoll (Ficoll-Paque Plus, GE Healthcare) density gradient centrifugation. Cells were washed twice with PBS and resuspended in RPMI-1640^+^ (RPMI1640 Dutch modification supplemented with 10-µg/mL gentamycin, 2mM glutamax and 1mM pyruvate; Thermofisher).

PBMCs were plated in 96-well round-bottom plates (Corning) at a final concentration of 2.5 × 10^6^ cells/mL and in a total volume of 200 µL and stimulated with medium (negative control) or live *Aspergillus* at a final concentration of 1 × 10^7^/mL for 24 h. PBMCs in costimulation experiments were exposed to 10-µg/mL TriDAP (Invivogen) and subsequently stimulated with medium or live resting conidia (1 × 10^7^/mL). After stimulation, culture supernatants were collected and stored at −20°C until cytokine measurement. Cells were either analyzed for surface receptor expression by flow cytometry or assessed for the fungal killing capacity.

### Flow Cytometry

Surface pattern recognition receptor expression on human monocytes was assessed following stimulation of PBMCs with TriDAP as described above. Monocytes were stained with anti-human CD14 conjugated with FITC (BD) and anti-human CD45 conjugated with PE-Cy7 in combination with, anti-human CD282 (TLR2) Alexa647 (BD) and anti-human CD284 (TLR4) PE (Biolegend), or anti-human CD206 (Mannose Receptor) PE (Biolegend) and anti-human dectin-1 APC (R&D). CD14^+^ monocytes were gated within the population of CD45^+^ cells and subsequently, the mean fluorescence intensity (MFI) of TLR2, TLR4, Mannose receptor, and dectin-1 were assessed on the CD14^+^/CD45^+^ cells. For dectin-1 also a negative population was observed and the percentage of dectin-1^+^ cells was assessed in addition to the MFI. The cells were measured on an FC500 flow cytometer (Beckman Coulter) and the data were analyzed using CXP analysis software v2.2 (Beckman Coulter).

### *Ex Vivo* Stimulation of WT and *Nod1^−/−^* Murine Splenocytes and Bone Marrow-Derived Macrophages (BMDMs)

Wild-type and *Nod1^−/−^* C57Bl/6 mice were bred and maintained in the St. Jude Children’s Research Hospital, Memphis, TN, USA. Spleens were homogenized in 0.4-µM cell strainer (BD) and the cell number was adjusted to 1 × 10^7^/mL. The cell suspensions (500 µL/well) were placed in 24-well plates (corning) and incubated with culture medium or *Aspergillus* conidia for 1 or 5 days at 37°C and 5% CO_2_.

Bone marrow from mice (age between 8 and 20 weeks old) was flushed out after dissecting mouse legs. Differentiation into macrophages (BMDMs) occurred in 5 days at 37°C (5% CO_2_) in Dulbecco’s modified eagles medium (DMEM) supplemented with 30% of L929 supernatant containing 10% fetal bovine serum (HI, Invitrogen), 100-U/mL penicillin and 100-mg/mL streptomycin. The BMDMs (1 × 10^5^ /well) were placed in 96-well plates (corning) and incubated with culture medium or live *Aspergillus* conidia for 1 day at 37°C and 5% CO_2_. After stimulation, culture supernatants were collected and stored at −20°C until cytokine measurement.

### Silencing NOD1

Freshly isolated PBMCs were differentiated to macrophages using 6-day differentiation in 10% human serum (serum differentiated macrophages) or 10% human serum supplemented with 5-ng/mL GM-CSF (R&D Systems). After differentiation (1 × 10^5^) macrophages were seeded in 96-well plates and left for 2 h at 37°C to subsequently transfect them with 25-nM NOD1 siRNA (on target) or scrambled (non-targeted siRNA) control siRNA (smartpool, Thermo Scientific) for 48 h at 37°C (Dharmafect, Thermo Scientific). Subsequently, the culture medium was refreshed and cells were used for killing, ROS assays, and PCR analysis.

### Killing of *Aspergillus* by BMDMs, Human Macrophages, or PBMCs

Following differentiation, the mouse BMDMs (1 × 10^5^), human MDMs (1 × 10^5^), or freshly isolated PBMCs (5 × 10^5^) were exposed to *Aspergillus* conidia (2 × 10^6^) in 96-well plates a final volume of 200 µL. In several experiments dectin-1 was blocked using laminarin (100 µg/mL; Sigma Aldrich) or with a mouse dectin-1 blocking antibody (GE2; Thermo Fisher) or its isotype control. After 24 hat 37°C and 5% CO_2_, the cells were washed in water and plated in serial dilution on Sabouraud agar plates. CFUs were counted after 24 h incubation at 37°C.

### Quantitative Reverse Transcriptase PCR

RNA was isolated according to the protocol supplied with the TRIzol reagent. Isolated mRNA (1 µg) was reverse transcribed into cDNA using the iScript cDNA synthesis kit (BIORAD). Quantitative real-time PCR (qPCR) was performed using power SYBR Green PCR master mix (Applied Biosystems) and following primers for human samples hNOD1 Fwd 5′-AGAGGCTCTGCGGAACCA-3′ and Rev 5′-TGTGGAGATGCCGTTGGA-3′, hGAPDH Fwd 5′-AGGGGAGATTCAGTGTGGTG-3′ and Rev 5′-CGACCACTTTGTCAAGCTCA-3′ hCLEC7A Fwd 5′-ACAATGCTGGCAACTGGGCT-3′ and Rev 5′-GCCGAGAAAGGCCTATCCAAAA-3′ hTLR2 Fwd 5′-GAATCCTCCAATCAGGCTTCTCT-3′ and Rev 5′-GCCCTGAGGGAATGGAGTTTA-3′ and the following primer sets form mouse samples mClec7a Fwd 5′-AGGTTTTTCTCAGCCTTGCCTTC-3′ and Rev 5′-GGGAGCAGTGTCTCTTACTTCC-3′, mGapdh Fwd 5′-AGGTCGGTGTGAACGGATTTG-3′ and Rev 5′-TGTAGACCATGTAGTTGAGGTCA-3′. PCR was performed using an Applied Biosystems 7300 real-time PCR system using PCR conditions 2 min 50°C, 10 min 95°C followed by 40 cycles at 95°C for 15 s and 60°C for 1 min. The RNA genes of interest were corrected for differences in loading concentration using the signal of the housekeeping protein GAPDH.

### IκBa Phosphorylation

For analysis of NFκB signaling pathways, the BMDMs were sub-cultured in 12-well cell culture plates for 16 h, and stimulated with live *Aspergillus* spores at 25 MOI of infection for indicated times. Protein lysates were prepared using the lysis buffer (10-mM Tris–HCl, 150-mM NaCl, 1% Nonidet P-40, supplemented with protease and phosphatase inhibitor cocktails; Roche). Protein samples were denatured by boiling in sample loading buffer-containing SDS and 100-mM DTT for 5 min and separated in denaturing SDS-PAGE. Separated proteins were transferred to PVDF membranes and immunoblotted with rabbit antibodies against total IκBa, Phospho-IκBa. All antibodies were purchased from Cell Signaling followed by secondary anti-rabbit HRP antibodies (JacksonImmunoResearch Laboratories).

### Cytokine Measurements

The cytokine levels were measured using commercially available ELISA assays according to the protocol supplied by the manufacturer. IL-1β, TNFα, IL-17, and IL-22 assays were from R&D Systems and IFNγ was from Sanquin. Mouse IL-1β, TNFα, IL-6, KC, IL-17, IL-22, and IFNγ in splenocyte stimulations were measured using the Luminex multiplex platform (Millipore). In the *in vivo* experiments mouse IL-1β, TNFα, IL-6, KC, and G-CSF were measured using commercially available ELISA assays from R&D Systems according to the protocol supplied by the manufacturer.

### NOD1 Immunofluorescence Staining

CD14^+^ cells were isolated from PBMCs using magnetic bead isolation (MACS Miltenyi) according to the protocol supplied by the manufacturer. CD14^+^ cells (1 × 10^5^) were allowed to adhere for 1 h to 12-mm Ø glass coverslips. After adherence, the CD14^+^ monocytes were exposed for 30 min to FITC labeled *Aspergillus* conidia in a ratio of (5:1/conidia/CD14 cells), after which the cells were fixed in Methanol. NOD1 was stained using rabbit anti-NOD1 and secondary stained with Goat anti-rabbit IgG H/L Alexa594 (Invitrogen). The coverslips were mounted in Vectashield with DAPI (Vector Laboratories) and immunofluorescence was observed at 1,000× magnification using a Zeiss LSM510 confocal microscope (Carl Zeiss).

### Statistical Analysis

Data are presented as the mean ± SEM, or as scatterplots representing individual data points and a line indicating the median value of all the data obtained in experiments. Experiments were conducted at least twice and the number of biological replicates (mice/human donors) is indicated in the figure legends for each graph. Unless otherwise indicated the Mann–Whitney *U* test was used to determine statistical significant differences between experimental groups with *p* < 0.05 = *, *p* < 0.01 = **, *p* < 0.001 = ***, and *p* < 0.0001 = ****. All data were analyzed using Graphpad Prism v6.0.

## Results

### NOD1 Localizes to *Aspergillus*-Containing Phagosomes

**Figure 1:**
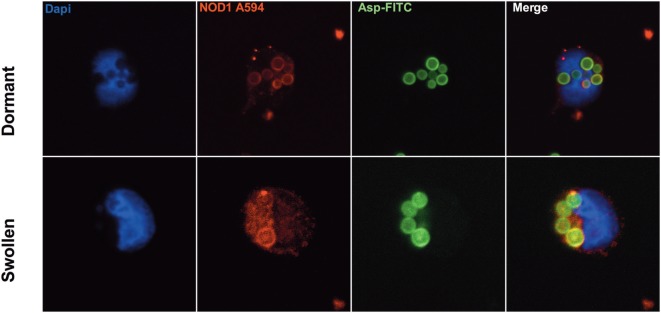
NOD1 localizing to *Aspergillus-*containing phagosomes. Representative confocal immunofluorescence images at 100× magnification demonstrating co-localization of NOD1 (stained with rabbit-anti-humanNOD1, conjugated with Goat-anti-RabbitIgG-Alexa594) with engulfed FITC-labeled dormant or swollen *Aspergillus fumigatus* spores in human monocytes (nuclear stain: DAPI).

Since NOD1 is an intracellular pattern recognition receptor for bacterial ligands, we wanted to investigate at which cellular level NOD1 interacts with *Aspergillus*. To assess the location of NOD1 during the interaction of monocytes with *Aspergillus*, the monocytes of healthy human volunteers were allowed to engulf *Aspergillus*, both resting and swollen conidia, for 1 h. Subsequently, NOD1 was stained by immunofluorescence staining. We observed that engulfed *A. fumigatus* resting or swollen conidia demonstrate a halo of NOD1 surrounding the conidia, suggestion colocalization to the phagosomes containing *Aspergillus* (Figure 1). In addition to the halo surrounding the conidia, a diffuse cytoplasmic staining of NOD1 could be observed.

### NOD1-Deficient Mice Do Not Develop IA

**Figure 2:**
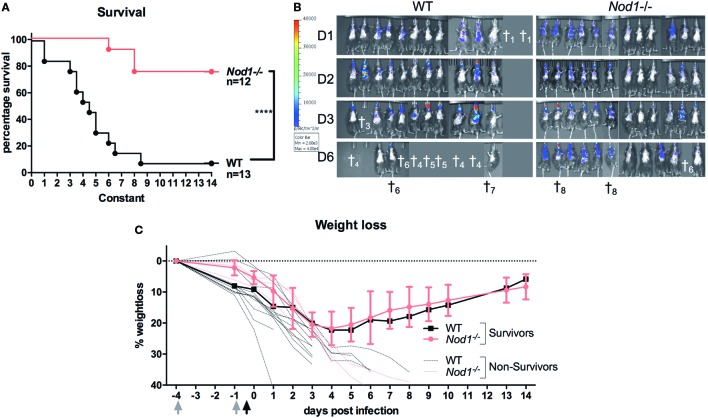
Immunocompromised *Nod1^−/−^* mice protected against invasive aspergillosis. Assessment of survival, fungal burden and weight in cyclophosphamide immunosuppressed wild-type (WT) (*n* = 13) and *Nod1^−/−^* (*n* = 12) mice infected intranasally with 5 × 10^4^ conidia in three separate experiments (WT:*Nod1^−/−^* 5:6; 3:3; 5:3). **(A)** Kaplan–Meier survival curve of WT (*n* = 13) and *Nod1^−/−^* (*n* = 12) mice. *P*-values of the Kaplan–Meier curve were determined using the log-rank test. Data represent the cumulative data of three separate experiments. **(B)** Bioluminescence imaging representing the fungal burden in the lungs of the mice during the course of the infection. **(C)** Representative graph of percentage weight loss of surviving mice in one of the experiments where survival of WT (*n* = 5; 4 died; *n* = 1 shown) and *Nod1^−/−^* (*n* = 6; 1 died; *n* = 5 shown) mice was compared.

To investigate whether NOD1 plays a role in the susceptibility to aspergillosis, we subjected WT C57Bl6 and *Nod1^−/−^* mice to lethal *Aspergillus* infection. Survival experiments were performed in mice immunosuppressed with cyclophosphamide and subsequently infected with the bioluminescent *Aspergillus* strain 2/7/1 (24). In contrast to WT mice, *Nod1^−/−^* mice showed a significant improvement in 14-day survival (Figure 2A). Nine out of 12 *Nod1^−/−^* mice survived, whereas 12 out of 13 WT mice did not survive the infection. Bioluminescence imaging of the luciferase-expressing *Aspergillus* within the mice suggests that *Nod1^−/−^* mice more efficiently clear the fungi from the lung, whereas WT mice developed a progressing infection as indicated by the increasing luminescence signal (Figure 2B). When comparing the weight loss of mice post-infection we observed that *Nod1^−/−^* mice and a single-surviving WT mouse started to recover their weight from day 4 post-infection (pi), whereas all other WT mice sharply declined in weight and succumbed to the infection and the three non-surviving *Nod1^−/−^* mice demonstrated a similar weight loss as WT mice (Figure 2C).

### Reduced Inflammation and Improved Fungal Clearance in Nod1-Deficient Mice

**Figure 3:**
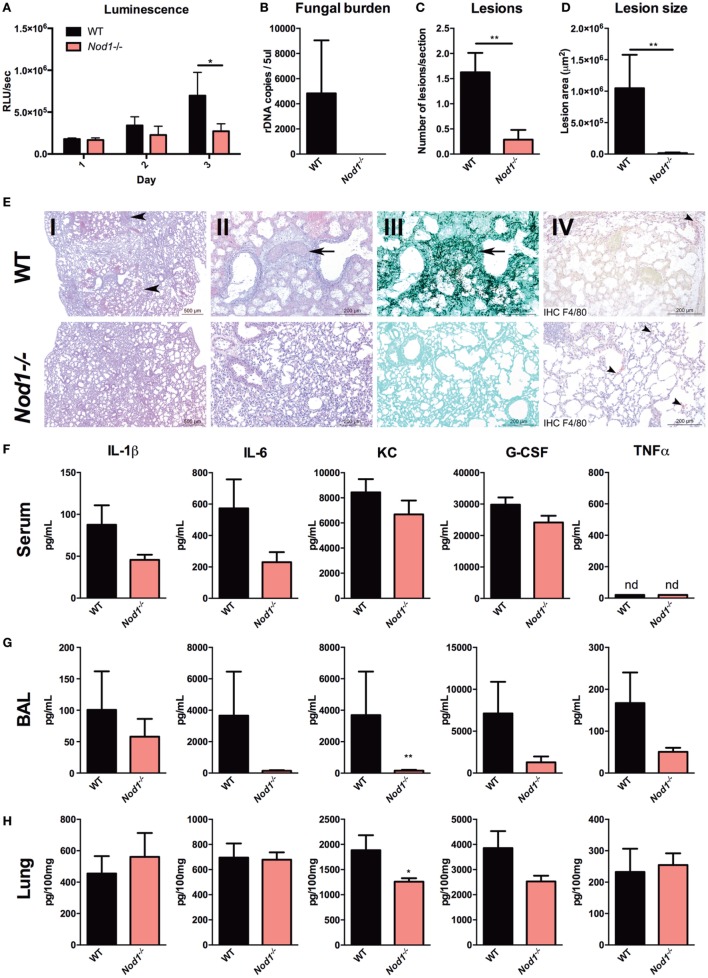
*Nod1^−/−^* mice reducing fungal burden, histological damage, and inflammation. Assessment of fungal burden, histopathological damage, and inflammation in *A. fumigatus*-infected wild-type (WT; *n* = 8) and *Nod1^−/−^* (*n* = 7) mice in two separate experiments (WT:*Nod1^−/−^* = 4:4 and 4:3). **(A)** Luminescence signal at day 1 to 3 post-infection revealing the fungal burden represented by the luminescence signal from live *Aspergillus* within infected WT and *Nod1^−/−^* mice. **(B)** Fungal burden as determined by amplification of *Aspergillus* ITS2 regions from lung homogenates. **(C–E)** Histology of lung sections of WT and *Nod1^−/−^* mice at day 3 pi, and morphometric analysis of the lesions in the whole lung sections using Image J software to quantify the lesions in **(C)** number and **(D)** size. Slides were stained by HE staining at [**(E)**; I] 2× and [**(E)**; II] 10x magnification, [**(E)**; III] Grocott’s Methenamine Silver staining at 10× magnification or [**(E)**; IV] immunohistochemistry with anti-F4/80 antibody counterstained with HE staining. **(F–H)** IL-1β, IL-6, KC, G-CSF, and TNFα levels in **(F)** serum, **(G)** broncheoalveolar lavage (BAL), and **(H)** lung homogenates measured at day 3 pi. Data are represented as mean ± SEM and means were compared using the Mann–Whitney *U* test. *P*-values of statistical tests are shown within the graphs.

To investigate differences in fungal burden, histological damage and inflammation in a standardized fashion, an experiment was performed where cyclophosphamide immunosuppressed mice were infected with the bioluminescent *Aspergillus* strain 2/7/1, but were sacrificed at day 3 pi. The luminescence signal from the lung reveals that *Nod1^−/−^* mice have a significantly reduced fungal burden compared with WT mice (Figure 3A). This observation could be confirmed by a quantitative *Aspergillus* PCR, which revealed the absence of *Aspergillus* DNA in the lung homogenates of *Nod1^−/−^* mice. However, in the lung homogenates of WT mice *Aspergillus* could be detected (Figure 3B). To assess how fungal burden correlates with pathological damage to the lungs, a histopathological analysis was performed. Morphometric analysis of the histology revealed significantly fewer lesions in the lung sections of *Nod1^−/−^* mice compared with WT mice (Figure 3C). Moreover, the size of the lesions affected a significantly smaller part of the lungs (Figure 3D). The morphometric analysis of pulmonary lesions corresponds with the finding that practically no fungi could be detected with Grocott methamine silver staining (Figure 3E, III). Based on immunohistochemistry for F4/80^+^ no differences in the presence of macrophages could be determined between WT and *Nod1^−/−^* mice (Figure 3E, IV). Systemic inflammation in the WT and *Nod1^−/−^* mice was assessed by measuring serum cytokine levels, and pulmonary inflammation was assessed by measuring cytokines in the BAL and in lung homogenates. Although *Nod1^−/−^* mice have a slight reduction in the levels of circulating proinflammatory cytokines, this was not significant compared with the WT mice (Figure 3F). In the BAL and lung homogenates, only a significant reduction in KC (CXCL1) levels were found when comparing *Nod1^−/−^* to the control group (Figures 3G,H, respectively). However, it must be noted that levels of other cytokines also tend to be lower in *Nod1^−/−^*, but due to a large variation in the control group the differences are not significant.

### Improved Cytokine Responses, Oxidative Burst, and Fungal Killing in Nod1 Deficient cells

**Figure 4:**
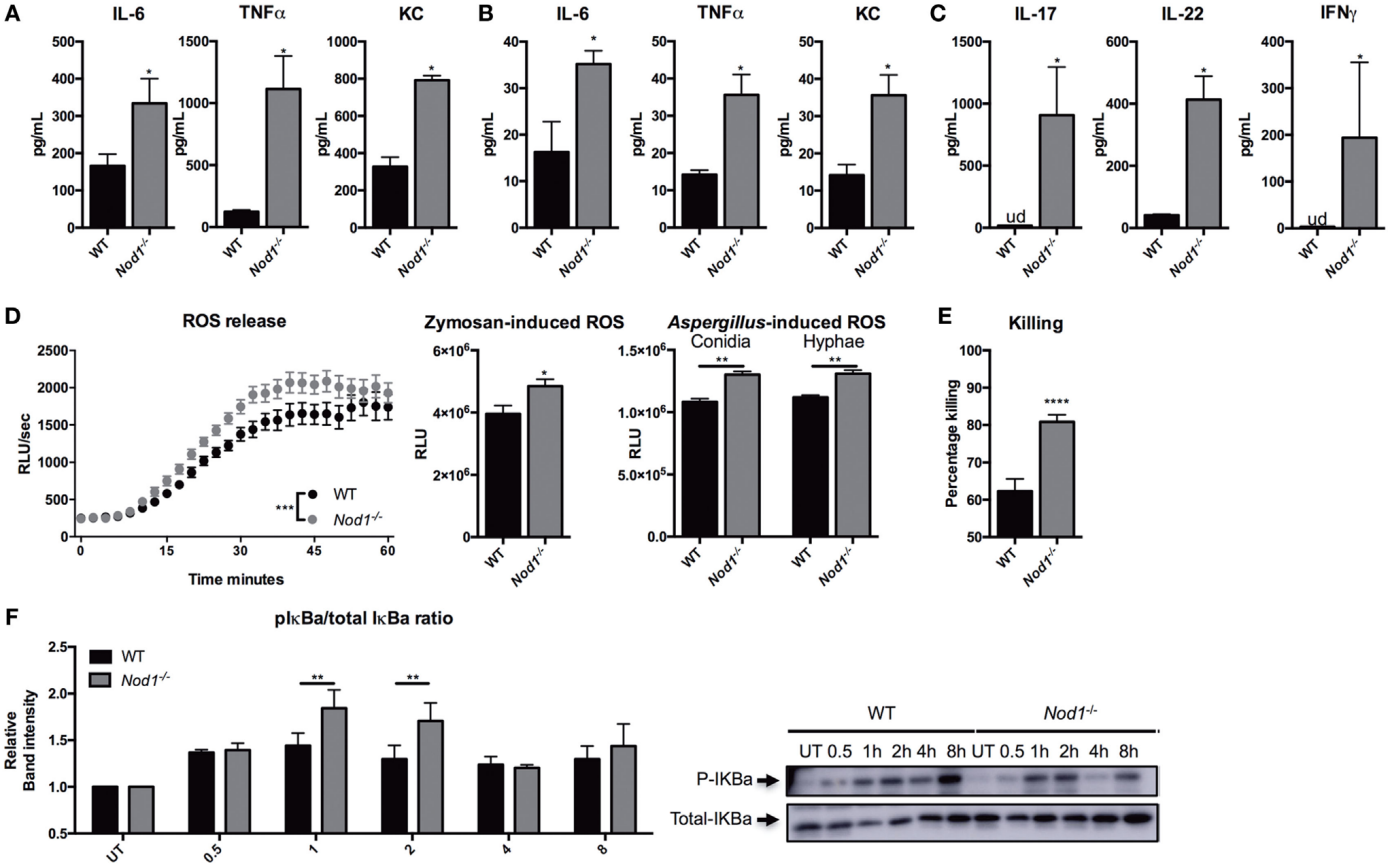
*Nod1*-deficient cells showing an augmented antifungal host response. **(A)** IL-6, TNFα, KC, in culture supernatants of bone marrow-derived macrophages (BMDMs) (1 × 10^5^) from wild-type (WT) and *Nod1^−/−^* mice (*n* = 6) that were stimulated for 24 h with heat inactivated *Aspergillus* conidia (2 × 10^6^). **(B)** IL-6, TNFα, KC, and **(C)** IL-17, IL-22, and IFNγ levels in culture supernatants of splenocytes (1 × 10^6^) from WT and *Nod1^−/−^* mice (*n* = 5 mice per group) that were stimulated for 5 days with heat inactivated *Aspergillus* conidia (2 × 10^7^). **(D)** ROS release by WT and *Nod1^−/−^* BMDMs following exposure to zymosan (*n* = 6). Time points were compared for significance by two-way ANOVA. Area under the curve of the ROS luminescence data of *Aspergillus* spores (*n* = 6) and swollen conidia (*n* = 6) (1 × 10^7^/mL) opsonized in 10% human serum and zymosan stimulated BMDMs. **(E)** CFU remaining of *A. fumigatus* plotted as percentage of input (2 × 10^6^) following exposure for 24 h to WT (*n* = 30) and *Nod1^−/−^* (*n* = 24) BMDMs (1 × 10^5^). **(F)** Representative Western Blot for phosphorylated and total IκBa in WT and *Nod1^−/−^* BMDMs following 0.5, 1, 2, 4, and 8 h of exposure to live *A. fumigatus spores*. IκBa phosphorylation measured as mean band intensity and corrected for total IκBa (*n* = 3). Data in bar plots are represented as mean ± SEM, data in scatter plots are represented as individual data points and median, and means were compared using the Mann–Whitney *U* test. ud = undetectable.

*Ex vivo* cytokine responses to *Aspergillus* were investigated in *Nod1*-deficient cells to identify the underlying mechanisms of the phenotypes observed in *Nod1^−/−^* mice. Cytokine responses by WT and *Nod1^−/−^* BMDMs were investigated. *Nod1^−/−^* BMDMs demonstrated significantly higher cytokine responses, compared with WT BMDMs (Figure 4A). Moreover, splenocytes were isolated from naive *Nod1^−/−^* and WT C57Bl/6 mice and stimulated with *Aspergillus*. Although the cytokine responses produced by splenocytes in response to *Aspergillus* were generally low, *Nod1^−/−^* splenocytes produced significantly more TNFα and KC in response to *Aspergillus* (Figure 4B). The *Aspergillus*-induced, T-helper cell cytokines IL-17, and IFNγ were undetectable (ud) and IL-22 was very poorly induced by WT splenocytes, while these cytokines were significantly elevated in culture supernatants of *Nod1^−/−^* splenocytes (Figure 4C). In addition to cytokine release, zymosan- and *Aspergillus-*induced ROS by BMDMs was significantly higher in *Nod1^−/−^* BMDMs (Figure 4D). The area under the curve was calculated to illustrate the quantitative difference in ROS release, with zymosan or *Aspergillus*. We also investigated whether this increased responsiveness of *Nod1^−/−^* BMDMs correlated with an altered capacity to kill *A. fumigatus* conidia. *Nod1^−/−^* BMDMs were significantly more efficient in killing *Aspergillus* conidia than WT BMDMs (Figure 4E). Subsequently, we investigated whether the differential cytokine induction and activation of *Nod1^−/−^* cells was due to differences in the capacity of these cells to activate NFκB signaling. BMDMs were exposed to live *Aspergillus* spores and subsequently lysed to assess IκBa phosphorylation as a marker for NFκB activation by Western Blot. WT macrophages show a steady increase in IκBa phosphorylation after stimulation, whereas the level of IκBa phosphorylation varies over time in *Nod1^−/−^* BMDMs with a significant increase after 1 and 2 h (Figure 4F).

### NOD1 Silencing Augments Oxidative Burst and Fungal Killing

**Figure 5:**
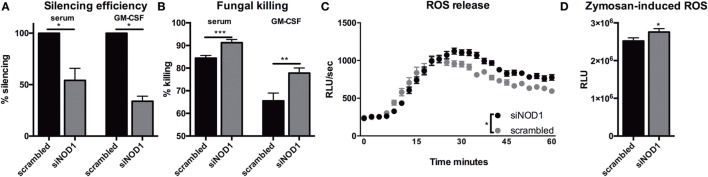
Silencing of NOD1 gene expression in human monocyte-derived macrophages improves fungal killing and oxidative burst. **(A)** Silencing efficiency of siRNA targeting NOD1(siNOD1) compared with scrambled siRNA in human macrophages (1 × 10^5^) differentiated in 10% serum (*n* = 6) or 10% serum with 5 ng/mL GM-CSF (*n* = 7). **(B)** CFU remaining of *A. fumigatus* plotted as percentage of input (2 × 10^6^) following exposure for 24 h to human macrophages (1 × 10^5^) differentiated in 10% serum or 10% serum with 5 ng/mL GM-CSF that were treated with scrambled siRNA or siNOD1. **(C,D)** ROS release by GM-CSF differentiated macrophages treated with scrambled siRNA or siNOD1following exposure to zymosan (*n* = 5). Time points were compared for significance by two-way ANOVA. Data in scatter plots are represented as individual data points and median. Means were compared using the Wilcoxon signed rank test. ud = undetectable.

Since *Nod1* deficiency impacts the killing capacity and ROS production in murine BMDMs, we validated these findings within a human background by silencing *NOD1* gene expression in human monocyte-derived macrophages (MDMs). *NOD1* silencing by siRNA targeting NOD1 (siNOD1) was confirmed by qPCR and a significant reduction of *NOD1* mRNA expression could be detected in both serum- and GM-CSF-differentiated MDMs (Figure 5A). Treatment with siNOD1 increased the killing capacity of MDMs when compared with cells that were transfected with scrambled siRNA (Figure 5B). ROS release was undetectable in the serum-differentiated MDMs; however, in GM-CSF-differentiated MDMs treated with siNOD1 the capacity to induce an oxidative burst was also slightly, yet significantly increased (Figures 5C,D).

### NOD1 Signaling Suppresses Fungal Killing Capacity

**Figure 6:**
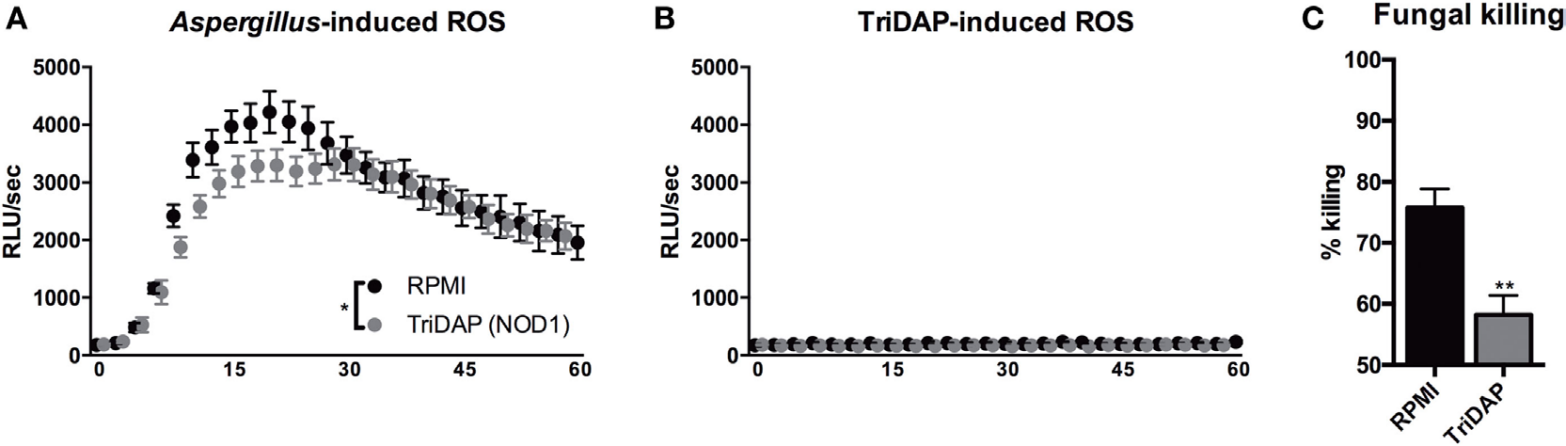
NOD1 activation dampening protective antifungal effector functions in human cells. **(A,B)** ROS release by human PBMCs following exposure to **(A)** Zymosan, when cells were pre-stimulated for 24 h in presence or absence of (10 µg/mL) TriDAP (*n* = 5), or **(B)** culture medium or (10 µg/mL) TriDAP alone (*n* = 3). **(C)** Fungal killing capacity of human GM-CSF differentiated monocytes-derived macrophages assessed as CFU remaining of *A. fumigatus* plotted as percentage of input (2 × 10^6^) following exposure for 24 h to (1 × 10^5^), after 24-h pre-stimulation in the presence or absence of (10 µg/mL) TriDAP (*n* = 9). All plots represent mean ± SEM. Means were compared using the Wilcoxon signed rank test, except for ROS curves, which were compared for significance using two-way ANOVA.

Since we observed that NOD1 deficiency or silencing resulted in an increased capacity to eliminate *A. fumigatus* conidia, we investigated whether activation of NOD1 could thus have an inhibitory effect on the host response to *Aspergillus*. To assess the effect of NOD stimulation on oxidative burst, PBMCs were stimulated with TriDAP and subsequently exposed to zymosan. Oxidative burst induced by zymosan was also reduced by pre-stimulation with the NOD1 ligand (Figure 6A). NOD ligands could potentially induce an oxidative burst thereby exhausting the cells; however, we found no detectable oxidative burst induced by NOD ligands (Figure 6B). Monocytes were differentiated with GM-CSF into MDMs and exposed to the NOD1 ligand TriDAP. MDMs that were exposed to TriDAP demonstrated a significantly reduced killing capacity compared with control cells (Figure 6C).

### NOD1 Activation or Deficiency Modulates Expression of Dectin-1

**Figure 7:**
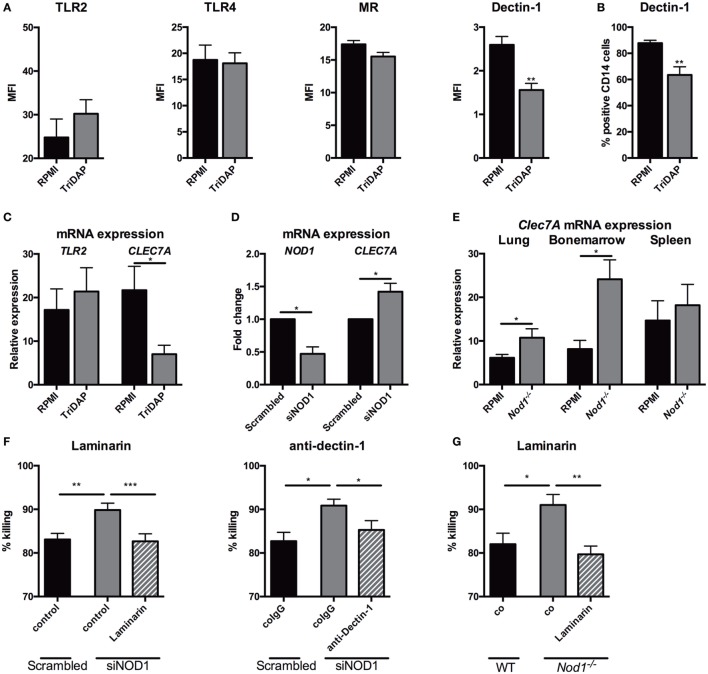
NOD1 suppresses dectin-1 expression and NOD1 deficiency augmenting fungal killing through dectin-1. **(A)** Surface expression of TLR2 (*n* = 6), TLR4 (*n* = 6), MR (*n* = 9) and dectin-1 (*n* = 9) on human CD14^+^ monocytes measured by flowcytometry following 24-h stimulation in the presence or absence of (10 µg/mL) TriDAP. **(B)** Percentage of dectin-1^+^ CD14^+^ monocytes following 24-h stimulation in the presence or absence of (10 µg/mL) TriDAP (*n* = 9). **(C)** mRNA expression of *TLR2* (*n* = 8) and *CLEC7A* (dectin-1) (*n* = 6) in human PBMCs following 24-h stimulation in the presence or absence of (10 µg/mL) TriDAP. **(D)** *NOD1* and *CLEC7A* (dectin-1) mRNA expression following siRNA treatment with scrambled siRNA or siRNA targeting NOD1(siNOD1) (*n* = 6). **(E)** *Clec7A* (dectin-1) expression in the lung, bone marrow and spleen of wild type (WT) (*n* = 8) and *Nod1^−/−^* (*n* = 6) at day 3 following *Aspergillus-*infection. Means were compared using the Mann–Whitney *U* test. **(F,G)** Fungal killing capacity assessed as CFU remaining of *A. fumigatus* plotted as percentage of input (2 × 10^6^). **(F)** Fungi killed by human GM-CSF differentiated monocytes-derived macrophages treated with scrambled siRNA or NOD1 targeting siRNA, the latter in the presence or absence of Laminarin (100 µg/mL) or a dectin-1 blocking antibody. Means were compared using the Wilcoxon signed rank test. **(G)** Fungi were killed by murine BMDMs of WT and *Nod1^−/−^* mice, the latter in the presence or absence of laminarin. Means were compared using the Mann–Whitney *U* test. All plots represent mean ± SEM.

Nucleotide-oligomerization domain receptors are known to interplay with TLRs *via* their downstream kinase RICK, and in particular with TLR2 (28–31). NOD1 deficiency or stimulation of NOD1 could very well impact killing, cytokine release, and ROS *via* modulation of PRRs. Therefore, surface expression of several PRRs, known to be involved in host defense against *Aspergillus*, were assessed by flow cytometry on PBMCs. Stimulation with TriDAP did not significantly affect TLR4 and MR expression on monocytes. dectin-1, however, was differentially regulated by NOD1 stimulation with a decrease of its expression (Figure 7A). This observation was also reflected by the number of dectin-1 positive monocytes (Figure 7B). To validate whether the reduced dectin-1 surface expression was regulated on a transcriptional level, RNA expression of *CLEC7A* (the gene encoding dectin-1) was assessed. Similarly, a decreased dectin-1 (*CLEC7A*) expression was observed (Figure 7C). In addition, siRNA treatment with siNOD1 of MDMs resulted in an increased dectin-1 (*CLEC7A*) expression (Figure 7D). To assess whether *Nod1*-deficient mice have altered dectin-1 expression, RNA was isolated from the lung, spleen, and bone marrow and dectin-1 (*Clec7A*) expression was measured. Compared with wild-type mice, *Nod1*-deficient mice had significantly elevated *Clec7A* expression in the lung and bone marrow, while only a trend toward increased *Clec7A* expression was observed in the spleen (Figure 7E). To determine whether the augmented killing capacity of human MDMs in which NOD1 is silenced is due to a functional enhancement of dectin-1 we systematically blocked dectin-1 using laminarin and dectin-1-blocking antibodies. The augmented killing capacity of human macrophages treated with *NOD1* targeting siRNA was abolished by dectin-1 blockade using laminarin or anti-human dectin-1 (Figure 7F). Similarly, laminarin mediated blockade of dectin-1 reversed the augmented fungal killing of *Nod1^−/−^* BMDMs (Figure 7G).

## Discussion

PRRs regulate the induction of an effective host defense against *A. fumigatus* through recognition of molecules present on the fungal cell wall and induction of potent antifungal effector mechanisms (4, 32). However, little is known about receptors that have a direct inhibitory effects on the induction of antifungal effector mechanisms. Here we demonstrate that the intracellular pattern recognition receptor NOD1 plays an inhibitory role in host response against *A. fumigatus*. We observed that NOD1 activation reduces fungal killing and the induction of oxidative burst. Conversely, murine *Nod1*-deficient cells or human cells in which *NOD1* gene expression was silenced show augmented fungal killing, oxidative burst, and cytokine responses. Most striking, despite being immunocompromised, *Nod1^−/−^* mice were observed to be less susceptible to *Aspergillus* infection, with reduced fungal burden, and pathological damage to the lungs. Finally, we demonstrate that the activity of NOD1 is inversely correlated with dectin-1 expression, where NOD1 stimulation reduces the expression of dectin-1, while *NOD1* silencing in human macrophages or murine *Nod1* deficiency was associated with increased *CLEC7A* (dectin-1) mRNA expression.

It is rarely observed that deficiency of a receptor is associated with decreased antifungal effector mechanisms. *Tlr9^−/−^* mice were found to be less susceptible to *Aspergillus* infection with reduced fungal burden (33). However, why TLR9 deficiency is protective is difficult to understand since TLR9 stimulation by CpG enhances the capacity of DCs to induce protective Th1 responses (34). Modulation of TLR5 in THP-1 cells is shown to negatively impact killing of *Aspergillus* conidia, with silencing of *TLR5* gene expression associated with increased fungal killing and activation of TLR5 with reduced fungal killing (35), reduced fungal killing was also observed in neutrophils (36). This modulation of fungal killing is similar to our data with NOD1 activation or siNOD1 in MDMs. However, a mutation in *TLR5* leading to a stop codon was identified as a risk factor for aspergillosis (37). Other than these PRRs, we are not aware of other receptors that negatively impact host defense against *Aspergillus*.

Following an otherwise lethal *Aspergillus* infection, *Nod1*-deficient mice demonstrated rapid fungal clearance, which was associated with an almost complete absence of pathological damage and fungal outgrowth in the lungs. In contrast, WT mice succumbed to the infection with severe fungal outgrowth in the lungs and significant pathological damage detected by histopathology. In contrast to our aspergillosis model, the NOD1 receptor is non-redundant in numerous bacterial infection models, such as *Mycobacterium tuberculosis* (9), *Pseudomonas aeruginosa* (38), *Shigella flexineri* (39), and *Helicobacter pylori* (40). In these models, NOD1 was required for an efficient cytokine response (38, 39) and killing of the pathogen (9, 38, 40). In contrast to these latter studies with bacteria, our data suggest that NOD1 has an inhibitory role on the antifungal host defense against *Aspergillus*. *Nod1* deficiency results in an increased capacity of BMDMs to kill live *Aspergillus* and an enhanced oxidative burst upon stimulation with zymosan. Strikingly, we also observe increased cytokine responses and enhanced NFκB translocation in murine *Nod1*-deficient cells. This is in contrast to a previous study that shows NOD1 to be required for NFκB translocation in the response of corneal epithelial cells to *A. fumigatus* (6). The fact that we observe similar results when we silence *NOD1* gene expression in human MDMs validates that the observed effects are due to the absence of NOD1. In contrast, we observed that NOD1 activation has the opposite effect of *NOD1* deficiency and silencing. Taken together, these data suggest that NOD1 inhibits crucial pathways in recognition of *Aspergillus* that limits the induction of protective antifungal effector mechanisms.

Mechanistically, we were able to demonstrate that activation of the NOD1 receptor by its ligand TriDAP reduces surface expression of the C-type lectin receptor dectin-1 on human monocytes, one of the most crucial receptors in host defense against *Aspergillus* (41–51). We found that the reduced surface expression was the result of a downregulation of *CLEC7A* mRNA expression when human monocytes were stimulated with the NOD1 ligand. Contrariwise, NOD1 silencing increased *CLEC7A* mRNA expression. Therefore, the activity of NOD1 seems to show a reverse correlation with *CLEC7A* transcription. Extending this to the *in vivo* model we observed increased *Clec7A* mRNA levels in the lungs and bone marrow of *Nod1^−/−^* mice, compared with WT controls. Dectin-1 is crucial for the induction of ROS by *Aspergillus*, which is in line with our data showing increased ROS by *Nod1*-deficient murine BMDMs or in human MDM where *NOD1* gene expression was silenced, which express more dectin-1 (52). We were able to pinpoint that the increased *dectin-1* expression, in the absence of NOD1, was responsible for augmented fungal killing by *Nod1^−/−^* BMDMs and human MDM in which NOD1 was silenced, as blockade of dectin-1 reversed the augmented killing.

ROS is essential for the host defense against *Aspergillus* and its importance is illustrated by patients with chronic granulomatous disease who are highly susceptible to infections with *Aspergillus* due to a defect in NADPH-dependent ROS production (53, 54). *Aspergillus* and Zymosan, which are used in our study to study the oxidative burst by murine and human macrophages, are both recognized by dectin-1 (55). We suggest that the modulation of dectin-1 expression by NOD1 could be the responsible mechanism for alterations in the capacity to induce an oxidative burst. Similarly we found that *Nod1^−/−^* BMDMs and human MDMs wherein *NOD1* gene expression was silenced have an increased capacity to kill conidia and a decreased conidial killing was observed in human MDMs when NOD1 was stimulated. These changes in conidial killing can also be explained by the differences in dectin-1 expression, as dectin-1 expression is required for efficient phagocytosis (45, 50, 51) and killing of *A. fumigatus* (48, 49) [reviewed in Ref. (42)].

In our *in vitro* studies, we observed that the absence of NOD1 improved fungal killing through enhancement of dectin-1 expression in BMDMs or human MDMs. Although it is evident that in host defense against *A. fumigatus* these cells employ dectin-1 to induce their antifungal effector functions, it is becoming increasingly evident that other cells also use dectin-1 to recognize *Aspergillus*. For example, the role of the pulmonary epithelium is an important tissue that must be taken into account, since these cells can also play an important role in anti-Aspergillus host defense. Dectin-1 on bronchial epithelial cells plays a role in the induction of innate immune responses to *Aspergillus* including the release of antimicrobial peptides such as defensins (51). Moreover, it has been demonstrated that enhancing dectin-1 on only the pulmonary epithelium promotes the resistance to IA (52). The role of dectin-1 in non-myeloid derived tissues is also highlighted by the observation that dectin-1 polymorphisms in the genotype of the recipients of hematopoietic stem cell transplants, which represent the non-myeloid tissues in the patient, predisposes to the development of aspergillosis (56). It cannot be concluded that the protection against aspergillosis that we observe in *Nod1^−/−^* mice is solely due to the increased dectin-1 expression on macrophages. We observed that dectin-1 expression in these mice is increased in both the bone marrow as well as the lung. Although resident macrophages in the lung could account for the changed dectin-1 expression, from our data it cannot be excluded that enhanced dectin-1 expression on the pulmonary epithelium does not play an additional role in the protection against *Aspergillus* infection.

Most interestingly, we were able to demonstrate that, in addition to its cytoplasmic expression, the NOD1 receptor localizes to *Aspergillus-*containing phagosome. Due to this localization to the phagosome, we suggest that NOD1 may also recognize fungal PAMPs that are exposed in the phagosome. Nevertheless, further studies are warranted to explore whether cytoplasmic sensing of fungal PAMPs or sensing of fungal PAMPs in the phagosome triggers the effects mediated by NOD1. Although NOD1 is crucial for recognition of bacterial cell wall products (57, 58) and activation of downstream protective immune mechanisms, we suggest that upon engagement of NOD1 with fungi, deleterious mechanisms are induced. Therefore, the potent protective effect of *Nod1* deficiency and beneficial effects of NOD1 silencing makes it tempting to suggest the blockade of NOD1 as a novel treatment strategy for IA. Currently, no pharmacological inhibitors are available to block NOD1 *in vivo*, but small molecule inhibitors that could potentially be used for therapy have been identified (59).

Collectively, we conclude that NOD1 induces a detrimental effect on protective antifungal mechanisms in host defense against *A. fumigatus*. The absence of NOD1 enhances the protective effector mechanisms such as cytokine production, oxidative burst, fungal killing, and dectin-1 expression. This observation paves the way for the development of new treatment strategies for IA that target NOD1.

## Ethics Statement

The human study was carried out in accordance with the recommendations of the guidelines for human research from the Arnhem-Nijmegen Medical Ethical Committee, with written informed consent from all subjects. All subjects gave written informed consent in accordance with the Declaration of Helsinki. The protocol was approved by the Arnhem-Nijmegen Medical Ethical Committee. The animal study was carried out in accordance with the recommendations of Institut Pasteur guidelines, in compliance with European animal welfare regulation and under regulations of the St. Jude Children’s Research Hospital Committee on Use and Care of Animals. The protocols were approved by the by the ethical committee for animal experimentation CETEA (Comité d’éthique en experimentation animale, Project license number 2013-0020) and by St. Jude Children’s Research Hospital Committee on Use and Care of Animals (protocol no 482-100265-1-/13), respectively.

## Author Contributions

MG, OI-G, and FV conceived and designed the experiments. MG, MJ, RM, OR, GJ, CF, WM, and OI-G performed the experiments. MG, MJ, RM, OR, GJ, WM, TK, and OI-G analyzed the data. TK provided valuable reagents. MG, AC, OI-G, and FV wrote the manuscript. MG, MJ, WM, TK, AC, OI-G, and FV amended the manuscript.

## Conflict of Interest Statement

The authors declare that the research was conducted in the absence of any commercial or financial relationships that could be construed as a potential conflict of interest.
