# Supplementary material for: The Absence of NOD1 Enhances Killing of Aspergillus fumigatus Through Modulation of Dectin-1 Expression
Source: Front Immunol. 2017 Dec 13;8:1777. doi: 10.3389/fimmu.2017.01777 (PMC5733348; doi:10.3389/fimmu.2017.01777)
Supplement: Supplementary file 1 [file Image_1.PDF]

Supplementary Figure 1

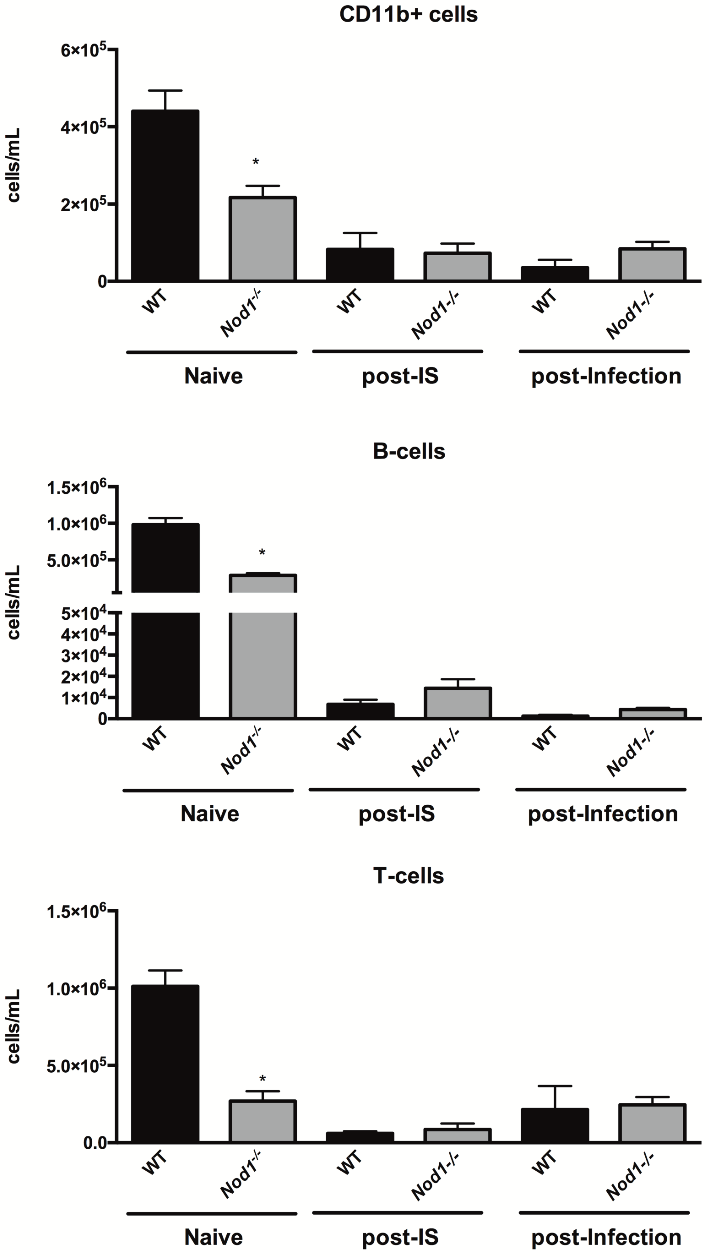

Supplementary figure 1

Number of circulating CD11b positive cells, B-cells and T-cells in wild-type and *Nod1*$^{-/-}$ mice. Samples were taken in Naïve mice, post cyclophosphamide immune suppression and 3 days post-infection.
