# Supplementary figures and images for: The Absence of NOD1 Enhances Killing of Aspergillus fumigatus Through Modulation of Dectin-1 Expression
Source: Front Immunol. 2017 Dec 13;8:1777. doi: 10.3389/fimmu.2017.01777 (PMC5733348; doi:10.3389/fimmu.2017.01777)

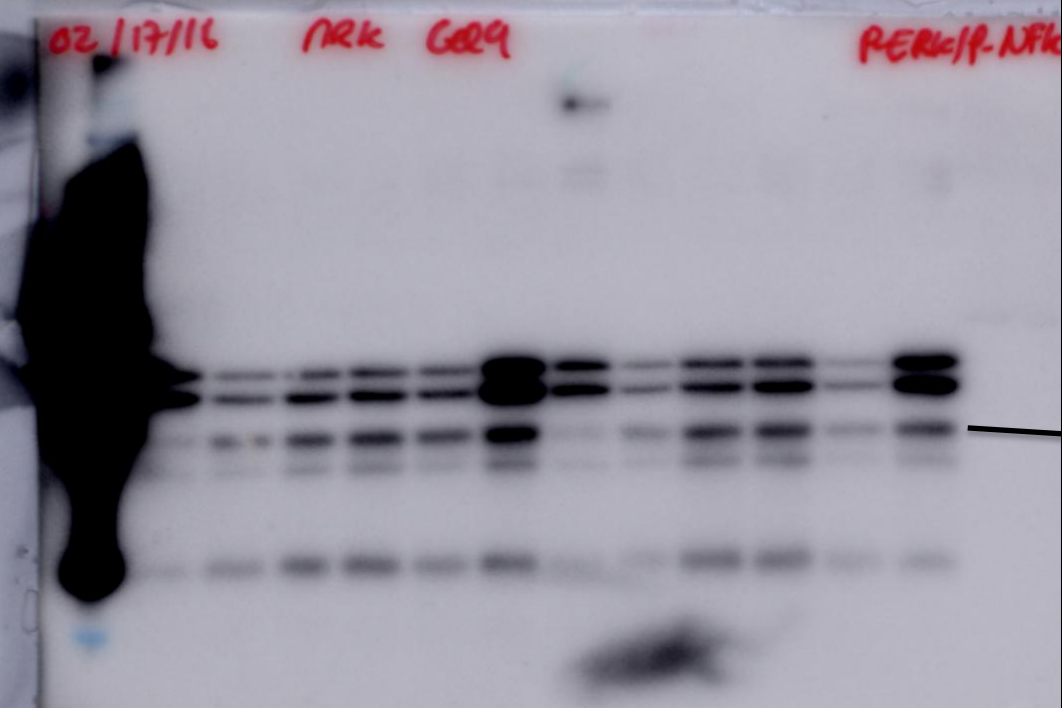

P-IKBa

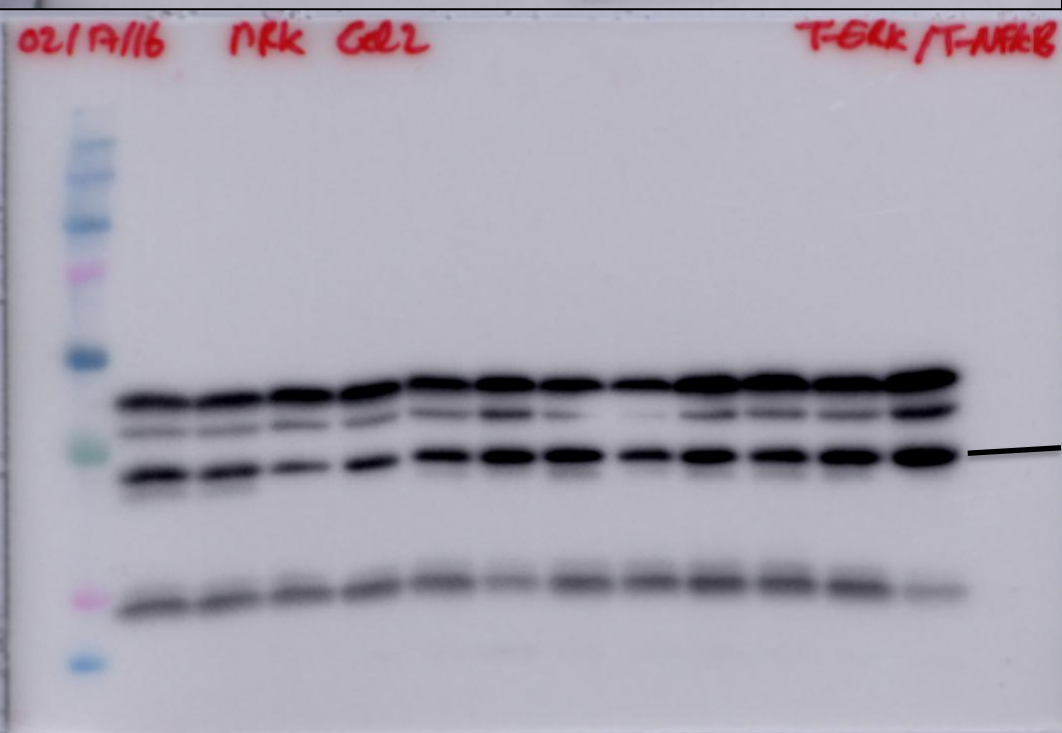

Total-IKBa

Supplement: Supplementary file 2 [file Image_2.PDF]
